# Extracorporeal carbon dioxide removal for treatment of exacerbated chronic obstructive pulmonary disease (ORION): study protocol for a randomised controlled trial

**DOI:** 10.1186/s13063-021-05692-w

**Published:** 2021-10-19

**Authors:** Tommaso Tonetti, Lara Pisani, Irene Cavalli, Maria Laura Vega, Elisa Maietti, Claudia Filippini, Stefano Nava, V. Marco Ranieri

**Affiliations:** 1 Department of Medical and Surgical Sciences (DIMEC), Alma Mater Studiorum – University of Bologna, Bologna, Italy; 2 Anesthesia and Intensive Care Medicine, Sant’Orsola Research Hospital, Bologna, Italy; 3 Department of Experimental, Diagnostic and Specialty Medicine (DIMES), Alma Mater Studiorum – University of Bologna, Bologna, Italy; 4 Pneumology and Respiratory Critical Care, Sant’Orsola Research Hospital, Bologna, Italy; 5 Department of Biomedical and Neuromotor Sciences (DIBINEM), Alma Mater Studiorum – University of Bologna, Bologna, Italy; 6 Dipartimento di Scienze Chirurgiche, Università di Torino, Torino, Italy

**Keywords:** Acute exacerbations of chronic obstructive pulmonary disease, COPD, Extracorporeal CO2 removal, ECCO2R, Non-invasive ventilation, NIV

## Abstract

**Background:**

Hypercapnic exacerbations are severe complications of chronic obstructive pulmonary disease (COPD), characterized by negative impact on prognosis, quality of life and healthcare costs. The present standard of care for acute exacerbations of COPD is non-invasive ventilation; when it fails, the use of invasive mechanical ventilation is inevitable, but is associated with extremely poor prognosis. Extracorporeal circuits designed to remove CO_2_ (ECCO_2_R) may enhance the efficacy of NIV to remove CO_2_ and avoid the worsening of respiratory acidosis, which inevitably leads to failure of non-invasive ventilation. Although the use of ECCO_2_R for acute exacerbations of COPD is steadily increasing, solid evidence on its efficacy and safety is scarce, thus the need for a randomized controlled trial.

**Methods:**

multicenter randomized controlled unblinded clinical trial including 284 (142 per arm) patients with acute hypercapnic respiratory failure caused by exacerbation of COPD, requiring respiratory support with NIV. The primary outcome is event free survival at 28 days, a composite outcome defined by survival in absence of prolonged mechanical ventilation, severe hypoxemia, septic shock and second episode of COPD exacerbation. Secondary outcomes are incidence of endotracheal intubation and tracheostomy, intensive care and hospital length-of-stay and 90-day mortality.

**Discussion:**

Acute exacerbations of COPD represent a significant burden in terms of prognosis, quality of life and healthcare costs. Lack definite evidence despite increasing use of ECCO_2_R justifies a randomized trial to evaluate whether patients with acute hypercapnic acidosis not responsive to NIV should undergo invasive mechanical ventilation (with all serious related risks) or be treated with ECCO_2_R to avoid invasive ventilation but be exposed to possible adverse events of ECCO_2_R. Owing to its pragmatic nature, sample size and composite primary outcome, this trial aims at providing valuable answers to relevant questions for clinical treatment of acute exacerbations of COPD.

**Trial registration:**

ClinicalTrials.gov, NCT04582799. Registered 12 October 2020, .

**Supplementary Information:**

The online version contains supplementary material available at 10.1186/s13063-021-05692-w.

## Background

Chronic obstructive pulmonary disease (COPD) is the third leading cause of death worldwide, resulting in a social and economic burden that is substantial and increasing [1, 2]. Hypercapnic exacerbations affect the prognosis (hospital mortality is approximately 10%) and quality of life (long-term outcome is poor) of patients with COPD [3, 4]. In addition, hypercapnic exacerbations of COPD have serious negative impacts on healthcare costs. Thus, prompt treatment of exacerbations may impact the clinical progression of COPD by ameliorating quality of life and prognosis [5, 6].

The pathophysiological hallmarks of COPD patients include expiratory flow limitation and small airway closure [7], making a prolonged expiratory time the only compensatory mechanism to maintain a stable tidal breathing. COPD exacerbations result in higher respiratory rates and reduced expiratory time, with decreased capacity of the respiratory muscles to generate pressure [7–9]. The consequent reduction of alveolar ventilation leads to a further worsening of CO_2_ retention and increased work of breathing [7].

The standard of care for patients with hypercapnic COPD exacerbations in the ICU is non-invasive ventilation (NIV) [10]. When NIV fails (i.e., arterial pH remains < 7.30), invasive ventilation through endotracheal intubation is initiated to restore adequate gas exchange. Patients requiring invasive mechanical ventilation have greater odds of death compared with patients successfully treated with NIV alone [4, 11].

Extracorporeal circuits designed to remove CO_2_ (ECCO_2_R) may add to improved efficacy of NIV by removing CO_2_ [12–15] and avoid worsening of respiratory acidosis [16–18]. Although available studies are limited to case series [12], several ECCO_2_R devices have been developed and proposed for the clinical use in patients with COPD [19]. These systems often represent modifications of renal replacement therapy circuits and are characterized by: (a) veno-venous by-pass systems; (b) extracorporeal blood flow of 0.3-0.5 liters/min; (c) 13 Fr bore catheters or a single co-axial catheter; (d) very low heparin doses or no heparin; (e) minimal volumes for circuit priming; (f) artificial membrane lung connected to a source of 100% O_2_ (usual flow 6-8 liters/min). These systems are able to reduce PaCO_2_ by 20-25% [20, 21]. These circuits have evolved in time and may be efficient even when using relatively small-bore catheters and low blood flows [19]. These modifications may reduce the side effects that have complicated earlier studies and underscore the rationale to perform this study.

### Trial hypothesis and main objective

A recent matched cohort study [14] with historical control, compared “*NIV-plus- ECCO*_*2*_*R*” and “*NIV-only*” in patients at risk of NIV failure, and showed that (a) the hazard of being intubated was three times higher in patients treated with “*NIV-only*” than in patients treated with “NIV-*plus*- *ECCO*_*2*_*R*”; (b) hospital mortality was significantly lower in “NIV *plus* ECCO_2_R” than in “NIV-*only*” [8% (95% CI 1.0-26.0%) vs. 33% (95% CI 18.0-57.5%), respectively]. However, ECCO_2_R-related complications were observed in almost half of the patients. A recent systematic review [12] evaluated the efficacy and safety of ECCO_2_R in patients with hypercapnic respiratory failure across 12 studies and showed that the majority of patients were either successfully weaned from mechanical ventilation or sustained on NIV, avoiding intubation. However, this high success rate was associated with a high frequency of potentially severe complications [12].

Accordingly, the main objective of this randomized multicenter clinical trial is to test the hypothesis that in patients with acute life-threatening exacerbation of COPD, use of ECCO_2_R could increase event-free survival as compared to standard of care. Event free survival is defined as survival at day 28 free of septic shock, second episode of COPD exacerbation, occurrence of severe hypoxemia, and prolonged mechanical ventilation.

## METHODS/DESIGN

### Trial design

This trial is a multicenter, prospective, randomized, controlled, unblinded clinical trial. The framework of the trial is superiority. The trial will include 284 patients with acute hypercapnic respiratory failure caused by exacerbation of COPD.

### Inclusion criteria

- Patients older than 18 and younger than 90 years
- Documented clinical history of COPD
- ICU admission for exacerbation of COPD requiring NIV support

After two hours of NIV at least two of the following criteria for high risk of NIV-failure [14, 22] must be fulfilled:

- arterial pH ≤ 7.25
- respiratory rate ≥30 breaths/min
- use of accessory muscles or paradoxical abdominal movements

### Exclusion criteria

- mean arterial pressure <60 mmHg despite infusion of fluids and vasoactive drugs
- ratio of arterial-to-inspired oxygen O_2_ fraction (PaO_2_/FiO_2_) ≤ 150 with FiO_2_ of less than 0.6 and PEEP of at least 5 cm H_2_O
- contraindications to anticoagulation (i.e. any of the following: platelet count <50,000/mm^3^; international normalized ratio (INR) >1.5; stroke or severe head trauma or intracranial arterio-venous malformation, or cerebral aneurysm, or central nervous system mass lesion within the previous 3 months; epidural catheter in place or expected to be positioned during the study; history of congenital bleeding diathesis; gastrointestinal bleeding within the 6 weeks prior to study entry; esophageal varices, chronic jaundice, cirrhosis, or chronic ascites; trauma);
- heparin-induced thrombocytopenia
- body weight >120 kg
- contraindication to continuation of active treatment
- failure to obtain consent
- difficult or impossible catheterization of femoral and jugular vein
- pneumothorax
- severe liver insufficiency (Child-Pugh scores >7) or fulminant hepatic failure
- diagnosis of acute or chronic neuromuscular disease
- chronic mechanical ventilation prior to hospital admission
- moribund patient
- inclusion in other interventional trials

### Primary outcome

The primary outcome is event free survival, defined as survival at day 28 free of any of the following: (a) prolonged mechanical ventilation; (b) development of septic shock; (c) occurrence of severe hypoxemia; (d) occurrence of a second episode of COPD exacerbation (either or not requiring mechanical ventilation); (e) 28-day all cause death. By analyzing the relevant epidemiological literature on COPD exacerbations [23–33] and on their complications [34–37] we could determine that these five elements are the most common complications in hospitalized AECOPD patients.

- **Prolonged mechanical ventilation** is defined as mechanical ventilation (invasive and non-invasive) applied for ≥ 14 days uninterrupted for more than 48 hours.
- **Septic shock** is defined by the presence of (a) sepsis (organ dysfunction caused by proven infection with an increase in the Sequential Organ Failure Assessment (SOFA) score of 2 points or more); (b) need for vasopressors to maintain a mean arterial pressure of 65 mmHg or greater; (c) serum lactate levels greater than 2 mmol/l (>18mg/dl) in the absence of hypovolemia.
- **Severe hypoxemia** is defined as occurrence of a ratio of arterial-to-inspired oxygen O_2_ fraction (PaO_2_/FiO_2_) ≤ 150 with FiO_2_ of less than 0.6 and PEEP of at least 5 cm H_2_O for at least 12 hours.
- **Second episode of COPD exacerbation** is defined as occurrence of sustained (≥24 h) increase in cough, sputum production, dyspnea and arterial pH≤7.30 (either or not requiring mechanical ventilation).
- **28-day all-cause mortality** is defined at 672 hours from randomization. All patients will be classified as either “alive at Study Day 28” or, if dead, “dead at Study Day 28.” For example, day zero is the day of randomization and day 1 is the next day and encompasses all events that occur midnight-to-midnight, etc.

### Secondary outcomes

Secondary outcomes are the following:

- Cumulative incidence of endotracheal intubation
- Cumulative incidence of tracheostomy
- Hospital length-of-stay
- ICU length-of-stay
- 90-day mortality
- Incidence of adverse events as defined in the ‘Safety’ paragraph (see below)

### Trial procedures

#### Informed consent

Informed consent will be taken by trained investigators. Before enrollment every patient must sign a written consent form for trial participation and for personal data processing. Anytime the patient is incapacitated and/or unable to fully understand the information provided, consent may be delayed (i.e., obtained after enrollment, as soon as the patient is again able to understand the information provided).

##### Randomization

Central randomization by blocks stratified only by center. The randomization is centralized: a random sequence stratified by center will be generated in order to guarantee a balancing of the arms within the center; the sequence will be implemented following a block algorithm. The random sequence will be generated by a server run by the coordinating center. Participant sites will obtain the allocation of each enrolled patient upon enrolment, after checking for inclusion/exclusion criteria.

##### Data collection

**Fig. 1:**
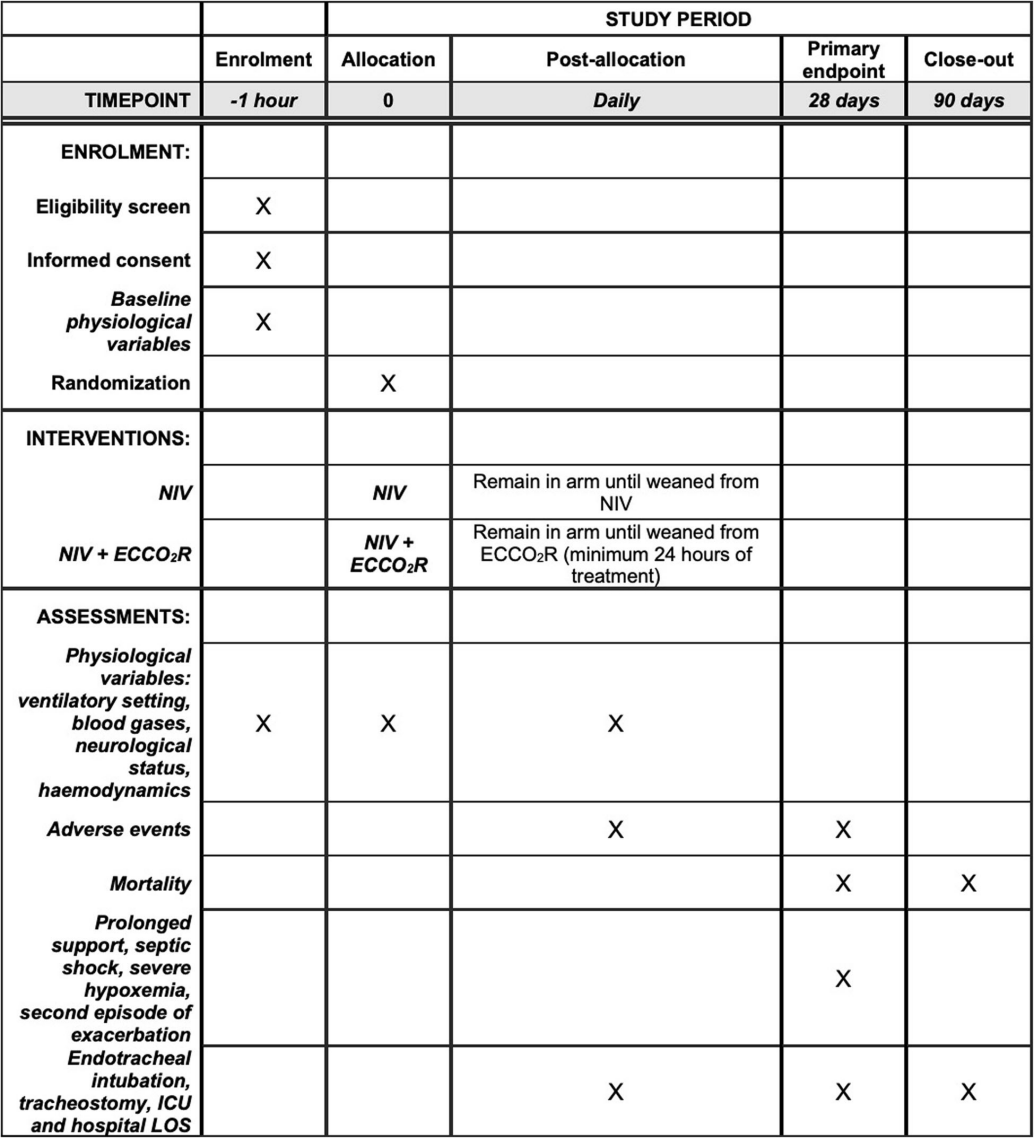
Schedule of enrolment, intervention and assessments

For eligible patients, study data will be collected after enrollment (Fig. 1). Study data will be collected and managed using REDCap electronic data capture tools hosted on a secure server at University of Bologna [38, 39]. Access to the study eCRF will be provided to all participating sites. All participant data will be stored anonymously through the use of codes. Participants’ identification data will be saved on an external memory, accessible only to study coordinators.

### Treatment of participants

#### Group A: Standard of care

NIV should be applied after a maximum of 2 hours from ICU admission using ICU ventilators with NIV-dedicated software. Use of full-face mask is recommended. Initial inspiratory positive airway pressure (IPAP) of 10 cmH_2_O and positive end-expiratory pressure (PEEP) of 5 cmH_2_O is recommended. IPAP may be then gradually increased by 2-5 cmH_2_O increments at a rate of approximately 5 cmH_2_O each 10 minutes until arterial pH>7.30 and patient tolerability has been reached. Inspiratory O_2_ fraction should be adjusted to maintain an O_2_ saturation of 88-92%.

Escalation to invasive mechanical ventilation should be considered by the attending physician (not involved in the study) when two of the following occur for at least 2 hours (‘urgency criteria’):

- respiratory rate >35 breaths/ min; arterial pH<7.25;
- PaCO_2_ greater than 60 mmHg;
- PaO_2_ less than 60 mm Hg with an FiO_2_ greater than 60%;
- respiratory arrest or signs of patient distress with accessory muscle recruitment and paradoxical abdominal or thoracic motion.

In addition, intubation will be immediately performed when any of the following is observed (‘emergency criteria’):

- hemodynamic instability (defined as 80-90 mmHg increase or a 30-40 mmHg decrease in systolic blood pressure relative to the baseline value or need for vasopressors to maintain systolic blood pressure > 85 mmHg or electrocardiographic evidence of ischemia or significant ventricular arrhythmias);
- need for sedation for major agitation (Richmond Agitation Sedation Scale ≥ 3) [40];
- acute neurological dysfunction (Kelly-Matthay score > 3) [41];
- cardiac arrest.

Patients should undergo a formal assessment of discontinuation from mechanical ventilation (invasive or non-invasive) and daily spontaneous breathing trials (SBT) will be performed to identify patients able to wean from mechanical ventilation once the cause of the exacerbation is adequately treated, the patient is hemodynamically stable (see above criteria for hemodynamic instability) and there are no signs of change in mental status (e.g., somnolence, coma, agitation, anxiety), onset or worsening of discomfort, diaphoresis, signs of increased work of breathing (use of accessory respiratory muscles, and thoracoabdominal paradox) *and* at least two of the following parameters are met:

- minute ventilation <15 l/min;
- PaO_2_/FiO_2_ ratio > 150 to 200; with PEEP ≤ 5 cmH_2_O
- FiO_2_ ≤ 0.4;
- pH > 7.30
- rapid shallow breathing index < 105 [42]

Pressure support ventilation (PSV) will be used to gradually decrease mechanical ventilatory support. Patient will be defined as “weaned from mechanical ventilation” (invasive or non-invasive) if the following 4 criteria are met for the last 30 minutes during a spontaneous breathing trial (SBT):

- SpO_2_≥90% and/or PaO_2_≥60 mmHg (PaO_2_ to take precedence if both available)
- Respiratory rate ≤30 bpm
- pH ≥ 7.35
- No respiratory distress (defined as 2 or more of the following):
  - Heart rate ≥ 120% of the 6:00 a.m. rate for ≥5 minutes
  - Significant use of accessory muscles
  - Abdominal paradox
  - Diaphoresis
  - Marked dyspnea as subjectively assessed by the investigator

#### Group B: Standard of care plus ECCO_2_R

NIV is applied and managed with the same modalities and at the same conditions as in Group A (standard of care).

After setting NIV, percutaneous vascular access is provided using a double lumen catheter inserted in the internal jugular vein or in the femoral vein (14F, Joline GmbH & Co. KG, Germany). An ECCO_2_R device will be used (DECAPsmart, Medica S.p.A.- Intensivecare s.r.l., Salerno, Italy). Blood flow is driven by a roller nonocclusive pump (0–450 mL/min) through a polymethylpentene artificial lung (priming volume, 200 mL; contact surface area, 0.8 m^2^; maximum blood flow rate, 450 mL/min) that is connected to a fresh gas flow source delivering 100% oxygen at a constant rate of 8 L/min. A starting dose of heparin (80 IU/kg bolus followed by ∼18 IU/kg/ hour infusion) will be delivered by using a syringe pump included in the system. Continuous infusion of heparin is hence titrated to maintain values of activated partial thromboplastin time (aPTT) in the range 35-80 sec or aPTT ratio of 1.5-2.0; aPTT is checked approximately every 2-3 hours during the first 6 hours from therapy initiation; afterwards, aPTT is checked every 6 hours. ECCO_2_R will be continued for at least 24 hours.

Intubation criteria are the same as in Group A (standard of care).

Weaning from ECCO_2_R must always precede weaning from mechanical ventilation. ECCO_2_R will be interrupted while keeping mechanical ventilation (invasive or non-invasive) when all of the following are achieved for at least 12 hours:

- respiratory rate<25 breaths/min;
- pH>7.35;
- arterial PCO_2_<20% of the baseline value;
- absence of use of the accessory muscles or paradoxical abdominal movements.

Criteria to (a) *run formal assessment of discontinuation* from mechanical ventilation (invasive or non-invasive) through a daily spontaneous breathing trial (SBT); (b) to be defined as “weaned from mechanical ventilation” (invasive or non-invasive) are the same as in Group A (standard of care).

Use of ECCO_2_R is stopped and device (and catheter) removed in the following situations:

- Air embolus in the extracorporeal circuit
- Circuit clotting leading to an abrupt stop of extracorporeal blood flow
- Device malfunction leading to abrupt stop of extracorporeal blood flow
- Severe thrombocytopenia (< 29,000/mm^3^)
- Fibrinogen < 1g/L or fibrinogen < 1.5 g/L with diffuse bleeding
- Anytime the physician in charge believes that the protocol may be compromising patient’s outcome

Interruptions of ECCO_2_R (for any cause) lasting longer than 6 hours are to be considered ‘treatment failure’ and lead to termination of the experimental protocol.

All concomitant therapies foreseen by the standard of care of enrolled patients are allowed.

Post-trial care will be delivered according to the standard of care for AECOPD patients.

### Safety

Safety will be assessed by measuring the frequency of serious adverse events (SAE). A serious adverse event (SAE) in the trial is defined as:

- any event that is fatal or immediately life threatening, permanently disabling, severely incapacitating or requires prolonged hospitalization **OR**
- any event that may jeopardize the patient and requires medical or surgical intervention to prevent one of the outcomes listed above **AND**
- which the attending physician perceives may be directly related to enrollment in the clinical trial.

Investigators will report severe adverse events to the clinical coordinator. An independent Data and Safety Monitoring Board (DSMB) will receive a detailed written report and evaluate whether the SAE is attributable to ECCO_2_R. SAE will be considered to be study-related if the event follows a reasonable sequence from a study procedure and could be produced by the study procedure. SAE will be considered not study related if they are thought to be primarily related to the underlying disease and its sequelae.

Other adverse events not fulfilling the above definition are recorded as ECCO_2_R related adverse events (ECCO_2_R-AE) and classified as:

- **mechanical**
  - difficulties upon catheter insertion (i.e. need for >2 attempts at catheter insertion during primary placement procedure);
  - pump malfunction (i.e. need for >2 consecutive interventions during the same treatment with the same blood circuit, regardless of the need for stopping the procedure or for replacement of the pump);
  - membrane lung clotting (leading to interruption of ECCO_2_R and/or circuit replacement);
  - catheter displacement;
  - cannula thrombosis;
  - air in the circuit;
  - tubing rupture;
  - system leaks;
- **clinical**:
  - hemolysis (i.e. serum free hemoglobin ≥ 100 mg/L or hematocrit reduction not related to hemorrhage or other causes of blood loss, jaundice, hemoglobinuria, impaired renal function);
  - bleeding (related to cannula insertion, and/or at cannula site; bleeding is considered “significant” if requires administration of ≥1 unit of packed red cells);
  - hemodynamic instability (see criteria in the ‘Treatment of participants’ paragraph);
  - ischemic/gangrenous bowel;
  - pneumothorax;
  - renal complications (i.e., occurrence of serum creatinine >1.5 mg/dl after initiation of ECCO_2_R);
  - infectious complications (i.e., occurrence of culture-proven new infection after initiation of ECCO_2_R);
  - thromboembolic complications (i.e., occurrence of deep venous thrombosis or pulmonary embolism after initiation of ECCO_2_R);
  - thrombocytopenia (platelet count below 50,000/mm^3^);
  - hypofibrinogenemia (i.e. fibrinogen < 1.5 g/L);
  - neurologic complications (i.e. occurrence of cerebral infarction, or clinical seizure, or cerebral hemorrhage or cerebral edema after initiation of ECCO_2_R).

Each SAE will be notified by the investigator to the coordinating center, DSMB and ethics committee within 24 hours of occurrence. A detailed report will be sent by the investigator within 5 days of occurrence. The DSMB will examine every report immediately and will take every measure needed to ensure patients safety. Moreover, the DSMB will also meet every 30 enrolled patients to perform all necessary safety analyses. The DSMB can irrevocably halt the trial if the data analysis would show risks for patients’ safety.

An appropriate insurance policy, covering all enrolled patients for possible harm will be activated before enrollment of the first patient.

### Sample size

A two-sided log-rank test with an overall sample size of 284 subjects (142 in the control group and 142 in the treatment group) achieves 90% power at a 0.05 significance level to detect a hazard ratio of 0.67 when the proportion surviving without events in the control group is 40% (we estimated a combined proportion of death, prolonged mechanical ventilation, septic shock, severe hypoxemia and second episode of COPD exacerbation of 60% [23–33]). The study will last 25 months of which 24 months to accrual and 1 month of follow up. No subject will drop out of the control/treatment group. We assume no cases of switch (crossing-over) between the arms: the proportion of the control group that switches to a group with a risk rate equal to the treatment group is equal to zero (and vice versa).

This study has been designed to demonstrate a relative increase of 35% in the event free survival (from 40% to 54%), assuming the above-mentioned parameters.

### Sites and sites monitoring

The study will be conducted at up to twenty sites within Italy. Three site visits for each center will be performed (start of enrollment, mid-study, end of enrollment). Additional site visits will be performed as required. The coordinating center (University of Bologna – Policlinico S.Orsola-Malpighi) will supervise all aspects of the study and will be available to participant sites 24 hours, 7 days. All sites must have significant experience in treating AECOPD and with ECCO_2_R (i.e., all centers must routinely use continuous renal replacement therapy and perform at least 10 ECCO_2_R runs per year). Frequent investigators’ meeting will be planned in order to motivate enrollment. The coordinating center will provide logistic support to all participating sites in order to ensure efficient enrolment.

More specific data about sites can be obtained from the corresponding author.

### Data and data monitoring

The study will be conducted in accordance with the current approved protocol. Independent study monitors will verify that the clinical trial is conducted, and data are generated, documented and reported according to the protocol and the current national and international regulations. Data quality will be assessed by the independent study monitors and frequent site visits (at least 3 visit/site/year) will be performed at each site, in order to ensure consistency throughout the duration of the trial.

### Access to data and publication

All data will remain property of the researchers and direct access to data will be granted only to authorized representatives of the regulatory authorities for inspective purposes.

Trial results will be communicated to relevant groups (participants, healthcare professionals, the public) via publication into peer-reviewed journal(s).

### Statistical analysis

Statistical analysis will be conducted according to the intention to treat principles (ITT), analyzing the whole sample size of all randomized patients.

Secondary analyses will be performed according to protocol adherence on “as treated” populations, with explorative and/or explicative purposes only.

No interim analyses will be performed. No predefined subgroup analysis will be performed.

#### Baseline characteristics

The baseline characteristics of the patients will be described by group, using the most appropriate statistics: quantitative variables will typically be summarized using frequencies and percentages for appropriate categorizations and may also be summarized using descriptive statistics. For variables summarized with descriptive statistics, the following will be presented: N, mean, standard deviation, median, q1, and q3. Categorical variables will be presented using frequencies and percentages.

No statistical comparisons between groups will be done on baseline variables.

#### Evaluation of the primary endpoint

EFS will be estimated using the Kaplan-Meier estimator applied to the ITT population, and compared between those who received NIV plus ECCO2R and those who did receive only NIV using the stratified log-rank test.

In addition, the Hazard Ratio (HR), along with 95% confidence intervals, will be estimated from a stratified Cox model with treatment group as a covariate.

#### Evaluation of secondary endpoints

- All components of the primary composite endpoint (singleton) will be examinated separately as independent proportion. The cumulative incidence of sepsis, hypercapnic respiratory failure, hypoxemic respiratory failure, prolonged mechanical ventilation >28days, tracheostomy and endotracheal intubation will be estimated with the Gooley method, to take into account the competitive risk of death, and compared with the Grey test. The Fine and Grey model will be used to estimate adjusted HR and their 95% CI. In addition, overall survival (OS) will be analyzed with Kaplan-Meier method and compared with the log rank test. A Cox proportional hazard model (if appropriate) will be used to estimate the Hazard Ratios (HR) and the 95% CI.
- Hospital length of stay and ICU length of stay will be described in each arm as median and interquartile range; they will be compared with the Wilcoxon rank sum test. The 95% confidence interval (CI) of the mean difference of measure will be estimated with a bootstrap method.
- Hospital mortality will be expressed as rate and a Chi square test will be used to evaluate the difference between the two groups.
- Incidence of adverse events and severe adverse events will be expressed as rate and a Chi square test will be used to evaluate the difference between the two groups.

## Discussion

The consistency of the data from systematic reviews and matched cohort studies [12–14], and the observation of the continuous increase use of ECCO_2_R despite the lack of solid evidence [12] confirm that the equipoise regarding the use of ECCO_2_R justifies our randomized clinical trial to evaluate whether patients with respiratory acidosis refractory to NIV should be intubated and take the risks associated with invasive mechanical ventilation, or should be connected to ECCO_2_R to avoid intubation, but run the risk of the potentially serious ECCO_2_R-related complications. Moreover, this trial may allow clinicians and hospital managers to know whether this treatment should be made available or should be avoided since inappropriate and associated to serious side effects.

One possible limitation of our study design is that it doesn’t include long-term patient-centered outcome measures; however, we feel that long-term outcome measures would add complexity to our pragmatic trial design and should be investigated in future trials, especially after clear evidence on shorter-term efficacy and safety are gathered.

The ORION trial, owing to its pragmatic nature, planned sample size and composite primary outcome is expected to provide valuable and definite answers to these important research questions. Hypercapnic exacerbation of COPD represent a significant clinical (prognosis), social (quality of life) and economic (health-care costs) burden worldwide and the avoidance of invasive mechanical ventilation through the application of ECCO_2_R (combined with NIV) may significantly impact the natural history of COPD itself.

Presently, the interest of the scientific community for the subject is very high, and this work is thus potentially highly significant in the field of respiratory intensive care medicine.

## Trial status

- Latest protocol version: 2.0, November 25^th^, 2019.
- At the time of writing, this trial is not yet recruiting participants.
- Estimate date for recruitment completion: December 31^st^, 2023.

## Supplementary Information

**Additional file 1.**

**Additional file 2.**

**Additional file 3.**

## Data Availability

Data sharing is not applicable to this article as no datasets were generated or analyzed during the current study.

## References

[1] GBD 2015 Mortality and Causes of Death Collaborators. Global, regional, and national life expectancy, all-cause mortality, and cause-specific mortality for 249 causes of death, 1980-2015: a systematic analysis for the Global Burden of Disease Study 2015. Lancet. 2016;388(10053):1459–544. 10.1016/S0140-6736(16)31012-1. Erratum in: Lancet. 2017;389(10064):e1. 10.1016/S0140-6736(16)31012-1 PMC5388903 27733281

[2] Mathers CD, Loncar D (2006). Projections of global mortality and burden of disease from 2002 to 2030. PLoS Med.

[3] Lindenauer PK, Stefan MS, Shieh MS, Pekow PS, Rothberg MB, Hill NS (2014). Outcomes associated with invasive and noninvasive ventilation among patients hospitalized with exacerbations of chronic obstructive pulmonary disease. JAMA Intern Med.

[4] Chandra D, Stamm JA, Taylor B, Ramos RM, Satterwhite L, Krishnan JA, Mannino D, Sciurba FC, Holguín F (2012). Outcomes of noninvasive ventilation for acute exacerbations of chronic obstructive pulmonary disease in the United States, 1998-2008. Am J Respir Crit Care Med.

[5] Vitacca M, Clini E, Rubini F, Nava S, Foglio K, Ambrosino N (1996). Non-invasive mechanical ventilation in severe chronic obstructive lung disease and acute respiratory failure: short- and long-term prognosis. Intensive Care Med.

[6] Murphy PB, Rehal S, Arbane G, Bourke S, Calverley PMA, Crook AM, Dowson L, Duffy N, Gibson GJ, Hughes PD, Hurst JR, Lewis KE, Mukherjee R, Nickol A, Oscroft N, Patout M, Pepperell J, Smith I, Stradling JR, Wedzicha JA, Polkey MI, Elliott MW, Hart N (2017). Effect of Home Noninvasive Ventilation With Oxygen Therapy vs Oxygen Therapy Alone on Hospital Readmission or Death After an Acute COPD Exacerbation: A Randomized Clinical Trial. JAMA.

[7] O'Donnell DE, Parker CM. COPD exacerbations. 3: Pathophysiology. Thorax 2006; 61:354-361, 4, DOI: 10.1136/thx.2005.041830. 10.1136/thx.2005.041830 PMC2104611 16565268

[8] Tobin MJ, Laghi F, Brochard L. Role of the respiratory muscles in acute respiratory failure of COPD: lessons from weaning failure, J Appl Physiol (1985). 2009;107:962–70. 10.1152/japplphysiol.00165.2009 19407256

[9] Laghi F, Goyal A (2012). Auto-PEEP in respiratory failure. Minerva Anestesiol.

[10] Rochwerg B, Brochard L, Elliott MW, Hess D, Hill NS, Nava S, Antonelli M, Brozek J, Conti G, Ferrer M, Guntupalli K, Jaber S, Keenan S, Mancebo J, Mehta S, Navalesi (members o P), Raoof (members o S) (2017). Official ERS/ATS clinical practice guidelines: noninvasive ventilation for acute respiratory failure. Eur Respir J.

[11] Alessandri F, Pugliese F, Mascia L, Ranieri MV (2018). Intermittent extracorporeal CO2 removal in chronic obstructive pulmonary disease patients: a fiction or an option. Curr Opin Crit Care.

[12] Sklar MC, Beloncle F, Katsios CM, Brochard L, Friedrich JO (2015). Extracorporeal carbon dioxide removal in patients with chronic obstructive pulmonary disease: a systematic review. Intensive Care Med.

[13] Kluge S, Braune SA, Engel M, Nierhaus A, Frings D, Ebelt H, Uhrig A, Metschke M, Wegscheider K, Suttorp N, Rousseau S (2012). Avoiding invasive mechanical ventilation by extracorporeal carbon dioxide removal in patients failing noninvasive ventilation. Intensive Care Med.

[14] Del Sorbo L, Pisani L, Filippini C (2015). Extracorporeal Co2 removal in hypercapnic patients at risk of noninvasive ventilation failure: a matched cohort study with historical control. Crit Care Med.

[15] Braune S, Sieweke A, Brettner F, Staudinger T, Joannidis M, Verbrugge S, Frings D, Nierhaus A, Wegscheider K, Kluge S (2016). The feasibility and safety of extracorporeal carbon dioxide removal to avoid intubation in patients with COPD unresponsive to noninvasive ventilation for acute hypercapnic respiratory failure (ECLAIR study): multicentre case-control study. Intensive Care Med.

[16] Abrams DC, Brenner K, Burkart KM, Agerstrand CL, Thomashow BM, Bacchetta M, Brodie D (2013). Pilot study of extracorporeal carbon dioxide removal to facilitate extubation and ambulation in exacerbations of chronic obstructive pulmonary disease. Ann Am Thorac Soc.

[17] Roncon-Albuquerque R, Carona G, Neves A, Miranda F, Castelo-Branco S, Oliveira T, Paiva JA (2014). Venovenous extracorporeal CO2 removal for early extubation in COPD exacerbations requiring invasive mechanical ventilation. Intensive Care Med.

[18] Pisani L, Fasano L, Corcione N, Comellini V, Guerrieri A, Ranieri MV, Nava S (2015). Effects of Extracorporeal CO2 Removal on Inspiratory Effort and Respiratory Pattern in Patients Who Fail Weaning from Mechanical Ventilation. Am J Respir Crit Care Med.

[19] Morelli A, Del Sorbo L, Pesenti A (2017). Extracorporeal carbon dioxide removal (ECCO2R) in patients with acute respiratory failure. Intensive Care Med.

[20] Duscio E, Cipulli F, Vasques F, Collino F, Rapetti F, Romitti F, Behnemann T, Niewenhuys J, Tonetti T, Pasticci I, Vassalli F, Reupke V, Moerer O, Quintel M, Gattinoni L (2019). Extracorporeal CO2 Removal: The Minimally Invasive Approach, Theory, and Practice. Crit Care Med.

[21] Morelli A, D'Egidio A, Orecchioni A, Alessandri F, Mascia L, Ranieri VM (2015). Extracorporeal co2 removal in hypercapnic patients who fail noninvasive ventilation and refuse endotracheal intubation: a case series. Intensive Care Medicine Experimental.

[22] Confalonieri M, Garuti G, Cattaruzza MS (2005). A chart of failure risk for noninvasive ventilation in patients with COPD exacerbation. Eur Respir J.

[23] Almagro P, Calbo E, Ochoa de Echaguen A (2002). Mortality after hospitalization for COPD. Chest.

[24] Patil SP, Krishnan JA, Lechtzin N, Diette GB (2003). In-hospital mortality following acute exacerbations of chronic obstructive pulmonary disease. Arch Intern Med.

[25] Ai-Ping C, Lee KH, Lim TK (2005). In-hospital and 5-year mortality of patients treated in the ICU for acute exacerbation of COPD: a retrospective study. Chest.

[26] Holguin F, Folch E, Redd SC, Mannino DM (2005). Comorbidity and mortality in COPD-related hospitalizations in the United States, 1979 to 2001. Chest.

[27] McGhan R, Radcliff T, Fish R, Sutherland ER, Welsh C, Make B (2007). Predictors of rehospitalization and death after a severe exacerbation of COPD. Chest.

[28] Waschki B, Kirsten A, Holz O, Müller KC, Meyer T, Watz H, Magnussen H (2011). Physical activity is the strongest predictor of all-cause mortality in patients with COPD: a prospective cohort study. Chest.

[29] Hoogendoorn M, Hoogenveen RT, Rutten-van Molken MP (2011). Case fatality of COPD exacerbations: a meta-analysis and statistical modelling approach. Eur Respir J.

[30] Almagro P, Cabrera FJ, Diez J, Boixeda R, Alonso Ortiz MB, Murio C, Soriano JB, Working Group on, COPD, Spanish Society of Internal Medicine (2012). Comorbidities and short-term prognosis in patients hospitalized for acute exacerbation of COPD: the EPOC en Servicios de medicina interna (ESMI) study. Chest.

[31] Mullerova H, Agusti A, Erqou S, Mapel DW (2013). Cardiovascular comorbidity in COPD: systematic literature review. Chest.

[32] Funk GC, Bauer P, Burghuber OC, Fazekas A, Hartl S, Hochrieser H, Schmutz R, Metnitz P (2013). Prevalence and prognosis of COPD in critically ill patients between 1998 and 2008. Eur Respir J.

[33] Klompas M, Anderson D, Trick W, Babcock H, Kerlin MP, Li L, Sinkowitz-Cochran R, Ely EW, Jernigan J, Magill S, Lyles R, O'Neil C, Kitch BT, Arrington E, Balas MC, Kleinman K, Bruce C, Lankiewicz J, Murphy MV, E Cox C, Lautenbach E, Sexton D, Fraser V, Weinstein RA, Platt R, CDC Prevention Epicenters (2015). The preventability of ventilator-associated events. The CDC Prevention Epicenters Wake Up and Breathe Collaborative. Am J Respir Crit Care Med.

[34] Chen CH, Lai CC, Wang YH, Wang CY, Wang HC, Yu CJ, et al. The Impact of Sepsis on the Outcomes of COPD Patients: A Population-Based Cohort Study. J Clin Med. 2018;7(11). 10.3390/jcm7110393. 10.3390/jcm7110393 PMC6262552 30373237

[35] Suissa S, Dell'Aniello S, Ernst P (2012). Long-term natural history of chronic obstructive pulmonary disease: severe exacerbations and mortality. Thorax.

[36] Hough CL, Caldwell ES, Cox CE, Douglas IS, Kahn JM, White DB, Seeley EJ, Bangdiwala SI, Rubenfeld GD, Angus DC, Carson SS, ProVent Investigators and the National Heart Lung and Blood Institute’s Acute Respiratory Distress Syndrome Network (2015). Development and Validation of a Mortality Prediction Model for Patients Receiving 14 Days of Mechanical Ventilation. Crit Care Med.

[37] Gungor S, Kargin F, Irmak I, Ciyiltepe F, Acartürk Tunçay E, Atagun Guney P, Aksoy E, Ocakli B, Adiguzel N, Karakurt Z (2018). Severity of acidosis affects long-term survival in COPD patients with hypoxemia after intensive care unit discharge. Int J Chron Obstruct Pulmon Dis.

[38] Harris PA, Taylor R, Thielke R, Payne J, Gonzalez N, Conde JG (2009). Research electronic data capture (REDCap)--a metadata-driven methodology and workflow process for providing translational research informatics support. J Biomed Inform.

[39] Harris PA, Taylor R, Minor BL (2019). The REDCap consortium: Building an international community of software platform partners. J Biomed Inform.

[40] Sessler CN, Gosnell MS, Grap MJ, Brophy GM, O'Neal PV, Keane KA, Tesoro EP, Elswick RK (2002). The Richmond Agitation-Sedation Scale: validity and reliability in adult intensive care unit patients. Am J Respir Crit Care Med.

[41] Kelly BJ, Matthay MA (1993). Prevalence and severity of neurologic dysfunction in critically ill patients. Influence on need for continued mechanical ventilation. Chest.

[42] McConville JF, Kress JP (2012). Weaning patients from the ventilator. N Engl J Med.

