# Supplementary material for: Extracorporeal carbon dioxide removal for treatment of exacerbated chronic obstructive pulmonary disease (ORION): study protocol for a randomised controlled trial
Source: Trials. 2021 Oct 19;22:718. doi: 10.1186/s13063-021-05692-w (PMC8524839; doi:10.1186/s13063-021-05692-w)
Supplement: Supplementary file 1 — Additional file 1. [file 13063_2021_5692_MOESM1_ESM.pdf]

SPRINGER NATURE

Change of authorship request form - Journals (pre-acceptance)

Important information. Please read.

- This form should be used by authors to request any change in authorship (adding/deleting authors) including changes in corresponding authors. This form should not be used for name changes. Please fully complete all sections. Use black ink and block capitals and provide each author's full name with the given name first followed by the family name.

- By signing this declaration, all authors guarantee that the order of the authors are in accordance with their scientific contribution, if applicable as different conventions apply per discipline, and that only authors have been added who made a meaningful contribution to the work.

- Please note, in author collaborations where there is formal agreement for representing the collaboration, it is sufficient for the representative or legal guarantor (usually the corresponding author) to complete and sign the Authorship Change Form on behalf of all authors, **next to the added/removed author(s)**. **(Complete Section 3, followed by Section 6.)**
  In author collaborations where there is no formal agreement for representing the collaboration and **there are more than 10 authors**, one may sign for all, provided the signer appends correspondence that attests that each of the authors have agreed to the change **and the added/removed authors sign the form**. **(Complete Section 3, followed by Section 6.)**

- Please note, we cannot investigate or mediate any authorship disputes. If you are unable to obtain agreement from all authors (including those who you wish to be removed) you must refer the matter to your institution(s) for investigation. Please inform us if you need to do this.

- If you are not able to return a fully completed form within **30 days** of the date that it was sent to the author requesting the change, we may have to withdraw your manuscript. We cannot publish manuscripts where authorship has not been agreed by all authors (including those who have been removed).

- Incomplete forms will be rejected.

- Please return/upload this form, fully completed, to the Journals Editorial Office. The Journal and/or Publisher will consider the information you have provided to decide whether to approve the proposed change in authorship. We may decide to contact your institution for more information or undertake a further investigation, if appropriate, before making a final decision.

SPRINGER NATURE Springer Nature is one of the world's leading global research, educational and professional publishers, created in May 2015 through the combination of Nature Publishing Group, Palgrave Macmillan, Macmillan Education and Springer Science+Business Media.

SPRINGER NATURE

Change of authorship request form - Journals (pre-acceptance)

Section 1: Please provide the current title of manuscript

Manuscript ID no.: TRLS-D-21-00032

Title: Extracorporeal Carbon Dioxide Removal for Treatment of Exacerbated Chronic Obstructive Pulmonary Disease (ORION): Study Protocol for a Randomised Controlled Trial

Section 2: Please provide the previous authorship, in the order shown on the manuscript before the changes were introduced. Please indicate the corresponding author by adding (CA) behind the name.

	First name(s)	Family name	ORCID or SCOPUS id, if available
1st author	Tommaso	Tonetti	0000-0001-9676-3595
2nd author	Lara	Pisani	0000-0001-9224-8158
3rd author	Irene	Cavalli	
4th author	Maria Laura	Vega	
5th author	Claudia	Filippini	0000-0003-0194-142X
6th author	Stefano	Nava	0000-0002-8404-2085
7th author	V. Marco	Ranieri (CA)	0000-0002-0427-1874
8th author			
9th author			
10th author			

Please use an additional sheet if there are more than 10 authors.

SPRINGER NATURE Springer Nature is one of the world's leading global research, educational and professional publishers, created in May 2015 through the combination of Nature Publishing Group, Palgrave Macmillan, Macmillan Education and Springer Science+Business Media.

Change of authorship request form - Journals (pre-acceptance)

Section 3: Please provide a justification for change. Please use this section to explain your reasons for changing the authorship of your manuscript, e.g. what necessitated the change in authorship? Please refer to the (journal) policy pages for more information about authorship. Please explain why omitted authors were not originally included and/or why authors were removed on the submitted manuscript.

Elisa Maietti, M.Sc. substantially contributed to the revision of the manuscript and matches the criteria for authorship.

Section 4: Proposed new authorship. Please provide your new authorship list in the order you would like it to appear on the manuscript. Please indicate the corresponding author by adding (CA) behind the name. If the Corresponding Author has changed, please indicate the reason under section 3.

	First name(s)	Family name (this name will appear in full on the final publication and will be searchable in various abstract and indexing databases)	Affiliated institute	E-mail address
1st author	Tommaso	Tonetti	Alma Mater Studiorum – University of Bologna	tommaso.tonetti@unibo.it
2nd author	Lara	Pisani	Alma Mater Studiorum – University of Bologna	lara.pisani@unibo.it
3rd author	Irene	Cavalli	Alma Mater Studiorum – University of Bologna	irene.cavalli@studio.unibo.it
4th author	Maria Laura	Vega	Alma Mater Studiorum – University of Bologna	laluvega@gmail.com
5th author	Elisa	Maietti	Alma Mater Studiorum – University of Bologna	elisa.maietti2@unibo.it
6th author	Claudia	Filippini	Alma Mater Studiorum – University of Bologna	claudia.filippini@unito.it
7th author	Stefano	Nava	Alma Mater Studiorum – University of Bologna	stefano.nava@unibo.it
8th author	V. Marco	Ranieri (CA)	Alma Mater Studiorum – University of Bologna	m.ranieri@unibo.it
9th author				
10th author				

Please use an additional sheet if there are more than 10 authors.

SPRINGER NATURE Springer Nature is one of the world's leading global research, educational and professional publishers, created in May 2015 through the combination of Nature Publishing Group, Palgrave Macmillan, Macmillan Education and Springer Science+Business Media.

SPRINGER NATURE

Change of authorship request form - Journals (pre-acceptance)

Section 5: Author contribution, Acknowledgement and Disclosures. Please use this section to provide a new disclosure statement and, if appropriate, acknowledge any contributors who have been removed as authors and ensure you state what contribution any new authors made (if applicable per the journal or book (series) policy). Please ensure these are updated in your manuscript - after approval of the change(s) - as our production department will not transfer the information in this form to your manuscript.

New acknowledgements:

None

New Disclosures (financial and non-financial interests, funding):

Competing interests
The authors declare that they have no competing interests.

Funding
Funding is provided by the Italian Ministry for University and Research through a grant (PRIN2017 – grant no. 2017J4BE7A)

New Author Contributions statement (if applicable per the journal policy):

VMR and SN are joint chief investigators, they conceived the study and led protocol development.
TT, LP, IC and EM contributed to study design and protocol drafting.
CF is the trial main statistician and methodologist.
All authors read and approved the final manuscript.

State 'Not applicable' if there are no new authors.

SPRINGER NATURE Springer Nature is one of the world's leading global research, educational and professional publishers, created in May 2015 through the combination of Nature Publishing Group, Palgrave Macmillan, Macmillan Education and Springer Science+Business Media.

SPRINGER NATURE

Change of authorship request form - Journals (pre-acceptance)

Section 6: Declaration of agreement. *All* authors, unchanged, new and removed *must* sign this declaration.
(NB: Please print the form, (docu)-sign and return/upload a scanned copy. Please note that signatures that have been inserted as an image file are acceptable as long as it is handwritten. Typed names in the signature box are unacceptable.) *** Please delete as appropriate. Delete all of the bold if you were on the original authorship list and are remaining as an author.**

	First name	Family name		Signature	Date
1st author	Tommaso	Tonetti	I agree to the proposed new authorship shown in section 4 [redacted]		29.09.2021
2nd author	Lara	Pisani	I agree to the proposed new authorship shown in section 4 [redacted]		29.09.2021
3rd author	Irene	Cavalli	I agree to the proposed new authorship shown in section 4 [redacted]		29.09.2021
4th authors	Maria Laura	Vega	I agree to the proposed new authorship shown in section 4 [redacted]		29.09.2021
5th author	Elisa	Maietti	I agree to the proposed new authorship shown in section 4 **/and the addition**[redacted]**of my name to the authorship list**[redacted]		29.09.2021
6th author	Claudia	Filippini	I agree to the proposed new authorship shown in section 4 [redacted]		29.09.2021
7th author	Stefano	Nava	I agree to the proposed new authorship shown in section 4 [redacted]		29.09.2021

SPRINGER NATURE Springer Nature is one of the world's leading global research, educational and professional publishers, created in May 2015 through the combination of Nature Publishing Group, Palgrave Macmillan, Macmillan Education and Springer Science+Business Media.

SPRINGER NATURE

Change of authorship request form - Journals (pre-acceptance)

	First name	Family name		Signature	Date
8th author	V. Marco	Ranieri	I agree to the proposed new authorship shown in section 4 [redacted]	[illegible]	29.09.2021
9th author			I agree to the proposed new authorship shown in section 4 **/and the addition/removal*of my name to the authorship list** /and the proposed change in corresponding author		
10th author			I agree to the proposed new authorship shown in section 4 **/and the addition/removal*of my name to the authorship list** /and the proposed change in corresponding author		

Please use an additional sheet if there are more than 10 authors.

In case of author collaborations with formal agreement:

	Name of consortium/consortia	First name	Family name		Signature	Date
Representative/legal guarantor				I agree to the proposed new authorship shown in section 4 **/and the addition/removal*of my name to the authorship list** /and the proposed change in corresponding author		

Both added/removed authors should complete the information in the first table under Section 6.

---- End of form ----

SPRINGER NATURE Springer Nature is one of the world's leading global research, educational and professional publishers, created in May 2015 through the combination of Nature Publishing Group, Palgrave Macmillan, Macmillan Education and Springer Science+Business Media.
