# Supplementary material for: Extracorporeal carbon dioxide removal for treatment of exacerbated chronic obstructive pulmonary disease (ORION): study protocol for a randomised controlled trial
Source: Trials. 2021 Oct 19;22:718. doi: 10.1186/s13063-021-05692-w (PMC8524839; doi:10.1186/s13063-021-05692-w)
Supplement: Supplementary file 2 — Additional file 2. [file 13063_2021_5692_MOESM2_ESM.pdf]

FOGLIO INFORMATIVO – PARTECIPAZIONE ALLO STUDIO
Versione 2.0 del 25/11/2019

TITOLO: "Rimozione extracorporea di anidride carbonica per il trattamento della BPCO riacutizzata: studio clinico randomizzato e controllato"
CODICE PROTOCOLLO: *studio ORION*
PROMOTORE: *Dipartimento di scienze mediche e chirurgiche (DIMEC), Università di Bologna*
CENTRO SPERIMENTALE: U.O Anestesiologia e Terapia Intensiva Polivalente-Direttore Prof. V. M. Ranieri. Azienda Ospedaliera Universitaria di Bologna, Policlinico Sant'Orsola-Malpighi.
SPERIMENTATORE PRINCIPALE: Prof. Vito Marco Ranieri
COLLABORATORI: *Dr.ssa Elisabetta Pierucci, Dr.ssa Ida di Giacinto, Dr. Tommaso Tonetti, Dr.ssa Irene Cavalli*

Gentile Paziente,

Le viene proposto di partecipare ad uno studio sperimentale. Questo documento Le fornisce tutte le informazioni sullo studio, che vogliamo Lei comprenda perfettamente prima di dare il Suo consenso. Non esiti a fare tutte le domande che riterrà necessarie qualora ci siano degli aspetti che non Le sono chiari.

1. **Introduzione**

La broncopneumopatia cronico ostruttiva (BPCO) è la terza principale causa di morte in tutto il mondo. Le riacutizzazioni influenzano la prognosi e la qualità di vita. La mortalità ospedaliera dei pazienti ricoverati per insufficienza respiratoria secondaria a BPCO riacutizzata è di circa il 10%

2. **Scopo dello studio**

Lo studio ha come obiettivo quello di valutare se nei pazienti con esacerbazione acuta di BPCO, condizione potenzialmente letale, l'utilizzo di rimozione extracorporea di anidride carbonica (ECCO2R) è in grado di aumentare la sopravvivenza libera da eventi rispetto allo standard di cura. Lo studio prevede l'arruolamento di 284 pazienti. Le stime di reclutamento sono di 14 pazienti per ciascuno dei 18 centri partecipanti. Ogni paziente verrà seguito per un tempo di 28 giorni.

3. **Perché sono stato scelto?**

Le stiamo proponendo di partecipare a questo studio perché Lei è affetto da una forma di insufficienza respiratoria acuta ipercapnica secondaria a riacutizzazione della sua patologia di base, la broncopneumopatia cronica ostruttiva (BPCO).

4. **Cosa mi succede se partecipo e cosa dovrò fare?**

Si tratta di uno studio randomizzato, ovvero nel quale, se deciderà di partecipare, potrà essere casualmente allocato nel gruppo della sola ventilazione non invasiva (NIV-only) o nel gruppo ventilazione non invasiva e rimozione extracorporea di anidride carbonica (NIV plus ECCO2R). Nel gruppo della sola ventilazione non invasivariceverà una ventilazione non invasiva attraverso una maschera nasale, oronasale o un elmetto. Nel gruppo ventilazione non invasiva-plus-rimozione extracorporea di anidride carbonica, in associazione alla maschera o elmetto per la ventilazione non invasiva , verrà associata una rimozione extracorporea di anidride carbonica. Tale metodica consiste nel rimuovere una quota di anidride carbonica attraverso un catetere di discrete dimensioni inserito in una vena del collo o dell'inguine attraverso il quale il sangue viene spinto costantemente verso una "membrana polmonare artificiale" (un filtro che aggiunge ossigeno e rimuove anidride carbonica dal sangue). Il sangue privato di unaquota di anidride carbonica, verrà reinfuso dallo stesso catetere del prelievo. La

durata del trattamento con il dispositivo di rimozione extracorporea di anidride carbonica può arrivare fino ad 8 giorni.

L'assistenza clinica che riceverà sarà la stessa sia nel caso in cui decida di partecipare allo studio sia nel caso di un suo rifiuto alla partecipazione. La partecipazione allo studio non comporta da parte sua spese aggiuntive, né rimborsi. La partecipazione allo studio è volontaria. Anche nel caso in cui decidesse di prendere parte allo studio, può decidere in qualsiasi momento di ritirarsi. Le verrà in ogni caso garantita l'assistenza clinica.

5. **Quali altri trattamenti sono disponibili per il mio disturbo?**
Attualmente il trattamento di elezione per la sua malattia e in caso di insufficienza respiratoria acuta secondaria a BPCO riacutizzata è la ventilazione meccanica non invasiva. Nonostante i miglioramenti delle nostre conoscenze riguardo questa tecnica, un numero considerevole di pazienti non risponde a tale trattamento e necessita di intubazione endotracheale e di ventilazione meccanica invasiva, quest'ultime gravate da alti tassi di complicanze e mortalità

6. **Quali sono i possibili rischi/effetti collaterali o svantaggi se partecipo allo studio?**
Nel caso in cui Lei decidesse di partecipare allo studio e venisse allocato casualmente nel gruppo ventilazione non invasiva-plus-rimozione extracorporea di anidride carbonica, tale trattamento potrebbe provocare scompenso cardiaco con insorgenza di tachicardia o aritmie o ipotensione tale da richiedere introduzione o incremento dell'uso degli specifici farmaci. Inoltre non si possono escludere emorragie, lesioni vascolari e complicanze infettive legate alla tecnica di posizionamento del catetere all'interno del vaso venoso e all'eparina presente nel circuito del dispositivo (trombocitopenia eparinoindotta). La probabilità che questi effetti collaterali si verifichino è del 12% per gli eventi più gravi (che comunque non metterebbero a rischio la sua incolumità) e del 34% per gli eventi più lievi. Verrà continuamente monitorato per ricevere assistenza nel caso questi eventi dovessero manifestarsi.
Il trattamento con il dispositivo di rimozione extracorporea di anidride carbonica potrà essere interrotto precocemente nel caso in cui si verifichino eventi avversi di natura meccanica (difficoltà con l'inserimento del catetere; malfunzionamento della pompa; coagulazione della membrana polmonare artificiale; spostamento del catetere; trombosi della cannula; aria nel circuito; rottura dei tubi; perdite del sistema) o di natura clinica (emolisi; sanguinamento significativo; sanguinamento correlato all'inserimento della cannula e/o nel sito della cannula; instabilità cardiocircolatoria; complicanze renali, intestinali, polmonari; complicanze infettive; complicanze tromboemboliche; complicanze neurologiche).

7. **Quali sono i possibili benefici?**
Il trattamento della ventilazione non invasiva più rimozione extracorporea di anidride carbonica (ECCO2R) potrebbe permetterci di ottenere una riduzione dei valori di anidride carbonica presenti nel suo sangue e conseguentemente un miglioramento delle sue condizioni cliniche, riducendo la durata di intubazione e di ventilazione invasiva, nonché la necessità di ricorrere alla tracheostomia.
E' tuttavia possibile che Lei non abbia alcun beneficio diretto per la sua patologia dalla partecipazione a questo studio, ma la sua partecipazione potrà contribuire a migliorare il trattamento e la comprensione di questa patologia in futuro.

8. **Che cosa succede se nuove informazioni diventano disponibili?**
Qualora nuovi significativi risultati, che possano influire sulla Sua volontà di partecipare allo studio, fossero resi disponibili nel corso dello studio stesso, Le verranno comunicati in maniera tempestiva.

9. **Che cosa succede se qualcosa va male?**
Il Promotore garantisce una polizza assicurativa a copertura dei danni cagionati ai soggetti dall'attività di questa ricerca per l'intero periodo della stessa e della responsabilità civile dello sperimentatore e del promotore, senza esclusione dei danni involontariamente cagionati in conseguenza di un fatto accidentale e/o imputabili a negligenza, imprudenza o imperizia.
La polizza (Lloyd's tramite broker assicurativo AON S.p.A Milano, codice identificativo di gara **6990408B29**) prevede un risarcimento danni per persona fino ad un massimo di Euro 1.000.000, con un massimale per il protocollo di Euro 10.000.000. La polizza non copre il valore eccedente tali massimali.
La polizza è operante per danni che si siano manifestati entro 36 mesi dalla data di conclusione della sperimentazione e la cui richiesta di risarcimento sia stata presentata entro 24 mesi dalla data di conclusione della sperimentazione.
Per qualsiasi domanda riguardante la garanzia di risarcimento e le cure mediche e per qualunque disturbo o danno Lei dovesse ritenere correlato alla sperimentazione, La preghiamo di contattare il Medico dello studio.

10. **Cosa succederà con i risultati dello studio?**
I risultati di questo studio potranno essere:
- presentati a convegni,
- essere pubblicati;
  essere utilizzati ai fini di loro valorizzazione e tutela o fonte di ulteriore ricerca (i dati raccolti verranno contraddistinti da un codice numerico e privi del suo nominativo per garantirne l'anonimato. I dati verranno trattati soltanto nella misura in cui sono indispensabili in relazione all'obiettivo dello studio. I dati raccolti verranno conservati per 10 anni e potranno essere utilizzati solo a scopo di ricerca).

11. **Chi è il Promotore dello studio?**
Dipartimento di scienze mediche e chirurgiche (DIMEC), Università di Bologna

12. **Chi ha valutato lo studio?**
Il Comitato Etico di Area Vasta Emilia Centro (CE AVEC) della Regione Emilia-Romagna ha valutato lo studio che verrà condotto secondo la normativa vigente in materia.

13. **Cosa succede se non volessi partecipare o se volessi ritirarmi dallo studio?**
Qualora Lei decidesse di non partecipare a questo studio o di interrompere la Sua partecipazione, è libero di farlo senza alcuna penalità o perdita di benefici e senza l'obbligo di fornire alcuna giustificazione. L'assistenza sanitaria Le verrà garantita indipendentemente dalla Sua partecipazione allo studio o eventuale volontà di interruzione.

14. **Per partecipare allo studio dovrò sostenere dei costi?**
Lei non dovrà sostenere nessuna spesa per la Sua partecipazione allo studio.

15. **Contatti per ulteriori informazioni**

Qualora avesse bisogno di ulteriori chiarimenti sullo studio o le sorgesse qualsiasi dubbio sulla partecipazione, può contattare lo Sperimentatore Responsabile dello studio e/o i suoi collaboratori:

Centro Sperimentale

U.O Terapia Intensiva Polivalente-Direttore Prof. V. M. Ranieri. Azienda Ospedaliera Universitaria di Bologna, Policlinico Sant'Orsola-Malpighi.

Sperimentatori

Dr.ssa Elisabetta Pierucci, Dr.ssa Ida Di Giacinto, Dr. Tommaso Tonetti, Dr.ssa Irene Cavalli

Telefono 0512143268 Fax 0516364333

Le verrà fornita una copia di questo foglio informativo e del modulo di consenso informato da conservare nel Suo archivio personale.

NOTE: 1 copia per il Paziente, 1 copia per lo Sperimentatore.

CONSENSO INFORMATO _CONSENSO ALLA PARTECIPAZIONE ALLO STUDIO

Versione 2.0del 25/11/2019

TITOLO:	"Rimozione extracorporea di anidride carbonica per il trattamento della BPCO riacutizzata: studio clinico randomizzato e controllato"
CODICE PROTOCOLLO: *studio ORION*	
PROMOTORE: *Dipartimento di scienze mediche e chirurgiche (DIMEC), Università di Bologna*	
CENTRO SPERIMENTALE: U.O Anestesiologia e Terapia Intensiva Polivalente-Direttore Prof. V. M. Ranieri.<br>Azienda Ospedaliera Universitaria di Bologna, Policlinico Sant'Orsola-Malpighi.	
SPERIMENTATORE PRINCIPALE: Prof. Vito Marco Ranieri	
COLLABORATORI: *Dr.ssa Elisabetta Pierucci, Dr.ssa Ida di Giacinto, Dr. Tommaso Tonetti, Dr.ssa Irene Cavalli*	

Il/La sottoscritto/a ______________________________

Nato/a a ______________________________ In data ______________

Residente a ______________________________

In via ______________________________

Dopo aver preso visione dell'informativa, **dichiara** quanto segue:

- di aver letto e compreso il Foglio Informativo per il Paziente (versione finale del 26/08/2019) per lo studio sopracitato e di aver avuto ampio tempo ed opportunità di porre domande ed ottenere risposte soddisfacenti dallo sperimentatore;
- di aver compreso che la partecipazione è volontaria e che potrà ritirarsi dallo studio in qualsiasi momento, senza dover dare spiegazioni e senza influenzare in alcun modo la sua futura assistenza medica;
- di aver compreso che parti della sua cartella, da cui risulti la partecipazione alla ricerca clinica, potranno essere visionate dalle Autorità Regolatorie, dal Comitato Etico e dall'Amministrazione Sanitaria Locale e, pertanto, acconsente che tali soggetti abbiano accesso ai suoi dati.

Conseguentemente alle sue dichiarazioni:

- **Accetta** di partecipare liberamente allo studio sopramenzionato.

ACCONSENTE ☐ NON ACCONSENTE ☐

che il suo Medico di famiglia sia informato che sta partecipando a questo studio.

ACCONSENTE ☐ NON ACCONSENTE ☐

all'utilizzazione dei Suoi dati/informazioni per fine di loro valorizzazione e tutela o fonte di ulteriore ricerca

Nome del Paziente	Data	Firma
Nome della persona che raccoglie il consenso informato	Data	Firma
Nome dello Sperimentatore	Data	Firma
Nome del testimone imparziale<br>(ove applicabile)	Data	Firma

Nota: 1 copia per il paziente, 1 copia per lo sperimentatore.
