# Supplementary material for: Extracorporeal carbon dioxide removal for treatment of exacerbated chronic obstructive pulmonary disease (ORION): study protocol for a randomised controlled trial
Source: Trials. 2021 Oct 19;22:718. doi: 10.1186/s13063-021-05692-w (PMC8524839; doi:10.1186/s13063-021-05692-w)
Supplement: Supplementary file 3 — Additional file 3. [file 13063_2021_5692_MOESM3_ESM.pdf]

Informazioni sul trattamento dei dati personali e particolari

**TITOLO STUDIO:**	"Rimozione extracorporea di anidride carbonica per il trattamento della BPCO riacutizzata: studio clinico randomizzato e controllato"
**PROTOCOLLO N.:**	ORION

Lei è invitato a prendere parte ad uno studio che si propone di verificare se nei pazienti con esacerbazione acuta di broncopneumopatia cronico ostruttiva (BPCO), condizione potenzialmente letale, l'utilizzo di rimozione extracorporea di anidride carbonica (ECCO2R) è in grado di aumentare la sopravvivenza libera da eventi rispetto allo standard di cura.
Lo studio sperimentale in oggetto verrà condotto presso l'U.O Anestesiologia e Terapia Intensiva Polivalente-Direttore Prof. V. M. Ranieri, Azienda Ospedaliera Universitaria di Bologna, Policlinico Sant'Orsola-Malpighi, in collaborazione con il Dipartimento di scienze mediche e chirurgiche (DIMEC), Università di Bologna. Le caratteristiche dello studio e le sue modalità di svolgimento sono descritte nel dettaglio nel documento "Foglio informativo- partecipazione allo studio".

L'Azienda Ospedaliera Universitaria di Bologna, Policlinico Sant'Orsola-Malpighi nonché l'Alma Mater Studiorum - Università di Bologna, quali autonomi Titolari del trattamento dei dati, tratteranno, ciascuno per gli ambiti di propria competenza, i suoi dati personali, in particolare quelli riguardanti la salute, esclusivamente in funzione della realizzazione dello studio e soltanto nella misura in cui siano indispensabili in relazione all'obiettivo dello studio, nel rispetto di quanto previsto dalla normativa vigente in materia di protezione dei dati personali, tra cui il Regolamento (UE) 2016/679 (Regolamento generale sulla protezione dei dati personali), il D.Lgs. 30 giugno 2003, n. 196 s.m.i. (Codice in materia di protezione dei dati personali) e conformemente alle misure di garanzia e a ogni altro provvedimento applicabile del Garante per la protezione dei dati personali.

SOGGETTI DEL TRATTAMENTO

Promotore:

- **Titolare del trattamento per finalità di avvio, gestione e monitoraggio della sperimentazione clinica, nonché di ricerca in ambito nazionale/internazionale**
  Alma Mater Studiorum – Università di Bologna (di seguito "Università") (sede legale: via Zamboni n. 33, 40126 - Bologna, Italia; e-mail: privacy@unibo.it; PEC: scriviunibo@pec.unibo.it)

- **Responsabile della protezione dei dati personali dell'Università**
  Sede legale: via Zamboni n. 33, 40126 - Bologna, Italia; e-mail: dpo@unibo.it; PEC: scriviunibo@pec.unibo.it;

Centro di Sperimentazione:

- **Titolare del trattamento per finalità di ricerca scientifica in ambito locale**

Azienda Ospedaliero-Universitaria di Bologna, Policlinico di S.Orsola (sede legale Via Albertoni n. 15, 40138 – Bologna, Italia;

- **Resposabile della protezione dei dati personali dell'Azienda USL di Bologna**
  Sede legale: via Albertoni 15, 40138 – Bologna, Italia;

FINALITÀ DEL TRATTAMENTO

I dati personali dell'Interessato e quelli appartenenti a particolari categorie (esempio i dati relativi al suo stato di salute) potranno essere trattati esclusivamente nell'ambito delle finalità di seguito riportate:

(A) Partecipazione allo studio e relative operazioni e attività connesse

Qualora l'Interessato presti il suo consenso a partecipare allo studio in oggetto, saranno raccolti alcuni dati che lo riguardano, mediante strumenti cartacei ed elettronici (in particolare, i dati saranno raccolti tramite una "scheda di raccolta dati", cosiddetta "CRF" di natura cartacea ed elettronica).

Il Centro di sperimentazione U.O Anestesiologia e Terapia Intensiva Polivalente e il promotore dello studio (Dipartimento di Scienze Mediche e Chirurgiche), ciascuno per gli ambiti di propria competenza e in accordo alle responsabilità previste dalla normativa vigente, tratteranno i dati personali dell'interessato (in particolare quelli sulla salute), soltanto nella misura in cui sono indispensabili in relazione all'obiettivo dello studio. In particolare, il Centro di sperimentazione compilerà una scheda clinica contenente alcune informazioni sulle condizioni cliniche del paziente e qualsiasi altra informazione connessa al trattamento del paziente. Il medico del Centro di Sperimentazione che seguirà lo studio identificherà l'interessato con un codice: i dati raccolti nel corso dello studio, ad eccezione del nominativo, saranno comunicati al promotore. Soltanto il medico ed i soggetti autorizzati dal Centro di Sperimentazione potranno collegare questo codice al Suo nominativo e conservare tale associazione per la durata di svolgimento dello studio.

I dati raccolti dal Centro di Sperimentazione (titolare della raccolta e conservazione dei dati identificativi a fini di ricerca scientifica), contraddistinti da un codice numerico e privi del suo nominativo, saranno quindi registrati in archivi informatici del Promotore, conservati per 10 anni e utilizzati esclusivamente per la realizzazione delle finalità sopra descritte.

I dati saranno conservati, unitamente al codice che identifica l'Interessato, presso il Centro di Sperimentazione sotto la responsabilità dell'U.O Anestesiologia e Terapia Intensiva Polivalente, Azienda Ospedaliera Universitaria di Bologna, Policlinico Sant'Orsola-Malpighi. Una volta scaduto il termine di conservazione sopra indicato, i dati verranno cancellati ovvero resi anonimi in modo che non sia più possibile risalire, in modo diretto o indiretto, all'identità dell'Interessato.

Entrambi i Titolari si impegnano a non trattare i dati particolari per fini diversi da quelli qui descritti e a non comunicarli o trasferirli a soggetti terzi, salvo che questi perseguano scopi scientifici direttamente collegati a quelli per i quali sono raccolti. I Titolari potranno comunicare o trasferire i dati dell'Interessato a enti e

istituti di ricerca, alle associazioni e agli altri organismi pubblici e privati aventi finalità di ricerca, esclusivamente nell'ambito di progetti congiunti.

Se l'Interessato accetta di prendere parte al presente studio, si informa che - oltre ai soggetti sopra indicati partecipanti alla ricerca – i dati potranno essere comunicati alle Autorità Regolatorie competenti, al Comitato etico indipendente di area vasta Emilia centro (CE-AVEC) e alle autorità sanitarie italiane che potranno esaminare tutta la documentazione sanitaria dell'Interessato raccolta nel corso dello studio: lo scopo di queste verifiche è controllare che la ricerca sia condotta correttamente e in conformità alle disposizioni vigenti e l'Interessato non avrà la facoltà di impedire tali comunicazioni.

(A1) Informazioni inerenti la diffusione

Si precisa che i dati saranno diffusi solo in forma rigorosamente anonima, ad esempio attraverso pubblicazioni scientifiche, statistiche e convegni scientifici.

(B) CONSERVAZIONE DEI DATI PER FUTURE ATTIVITÀ DI RICERCA

Considerato che nuove scoperte potrebbero indicare inedite opportunità di indagine ai ricercatori o consentire di effettuare ulteriori studi e ricerche sui dati particolari per lo studio di cui trattasi, l'Interessato può consentire la conservazione prolungata dei dati che lo riguardano in forma non anonima per un periodo di 10 anni dalla conclusione del presente studio, accettando di essere ricontattato dal Promotore e/o dal Centro di Sperimentazione affinché possa esprimere, se lo riterrà, un nuovo specifico consenso e autorizzare così una nuova ricerca sui propri dati.

Ove invece l'Interessato neghi il consenso al trattamento qui descritto, i dati verranno cancellati ovvero resi anonimi immediatamente allo scadere di termine di conservazione indicato al punto (A).

(C) Trattamento per finalità amministrative e contabili

I dati dell'Interessato saranno trattati per finalità amministrative/contabili e conservati per il tempo strettamente necessario al perseguimento di tali finalità, fatto salvo il termine di dieci anni per assicurare gli adempimenti fiscali, contabili e amministrativi richiesti dalla legge e salvo eventualmente termini più lunghi, non determinabili a priori, in conseguenza a diverse condizioni di liceità del trattamento (ad esempio azioni giudiziarie che rendano necessario il trattamento per oltre dieci anni).

ULTERIORI OPERAZIONI DI TRATTAMENTO

I dati potranno altresì essere trattati e/o comunicati a soggetti terzi, come di seguito specificato:

(i) Comunicazione al medico curante

L'Interessato può acconsentire che il Centro di Sperimentazione, in qualità di Titolare, informi il proprio medico curante della propria partecipazione allo studio in oggetto.

(ii) Comunicazione di risultati o notizie inattese

Nell'ambito della ricerca in oggetto, potrebbero essere conosciute alcune notizie inattese derivanti dai risultati dello studio.

Ove l'Interessato intenda conoscere i risultati della ricerca e dell'analisi, può esprimere il proprio consenso affinché gli vengano comunicate dette eventuali informazioni rilevanti. Ove invece l'Interessato neghi il consenso a tale finalità, si precisa che le informazioni relative a risultati della ricerca o notizie inattese verranno conservati nei limiti di tempo indicati al punto (A), salvo il caso in cui abbia acconsentito alla conservazione dei dati per un periodo più lungo (punto B). Nel caso in cui l'Interessato dovesse trovarsi in una condizione di impossibilità fisica, incapacità di agire o incapacità di intendere o di volere, le eventuali notizie inattese riguardanti lo stato di salute dell'Interessato potranno essere comunicate a chi esercita legalmente la legale rappresentanza, ovvero a un prossimo congiunto, a un familiare, a un convivente o unito civilmente ovvero a un fiduciario ai sensi dell'art. 4 della l. 219/2017 o, in loro assenza, al responsabile della struttura presso cui dimora.

BASE GIURIDICA DEL TRATTAMENTO E NATURA DEL CONFERIMENTO DEI DATI

La base giuridica dei trattamenti sopra descritti è da rinvenirsi nel consenso, ai sensi dell'art. 9, 2° comma, lett. a) del Regolamento. **Il consenso al trattamento per le finalità sopra descritte è libero e facoltativo e il suo mancato conferimento non preclude all'Interessato di accedere alle cure mediche richieste**.

Tuttavia, si precisa che il conferimento dei dati per le finalità di cui al punto (A) non è obbligatorio ma è indispensabile allo svolgimento dello studio. Il rifiuto di conferirli non consentirà all'Interessato di partecipare allo studio in parola. Si ribadisce che tale rifiuto non pregiudicherà in alcun modo eventuali cure o prestazioni sanitarie cui ha diritto.

Il conferimento dei dati ai fini di cui al punto (B) è facoltativo, cioè non discende da un obbligo normativo, ma è necessario per consentire la conservazione dei dati per un periodo più lungo di quello previsto per la conclusione del presente studio e per eventualmente consentire al Promotore e/o al Centro di Sperimentazione di ricontattare l'Interessato affinché possa esprimere, se lo riterrà, un nuovo specifico consenso e autorizzazione a una nuova ricerca. Il mancato conferimento dei dati per tali finalità avrà come unica conseguenza l'impossibilità di realizzare quanto da ultimo descritto.

Il conferimento dei dati ai fini di cui al punto (C) è necessario per ottemperare agli obblighi di legge in materia di conservazione dei documenti amministrativi e contabili. In tal caso la base giuridica è rinvenibile nell'art. 6, comma 1, lett. c) ed e), del Regolamento (UE) 2016/679.

DIRITTI DELL'INTERESSATO

Fatte salve le limitazioni all'esercizio dei diritti degli interessati di cui agli artt. 2-*undecies* e 2-*duodecies* del Codice in materia di protezione dei dati personali (D.Lgs.

196/03), l'Interessato partecipante al presente studio, può esercitare i diritti a lui riconosciuti ai sensi e nei limiti degli artt. 15-21 del Regolamento (UE) 2016/679, tra cui il diritto di chiedere l'accesso ai propri dati personali, la rettifica o la cancellazione degli stessi, nonché la limitazione del trattamento dei dati che lo riguardano, l'opposizione al trattamento e la portabilità dei dati.

Precisiamo inoltre che, ogni eventuale consenso manifestato dall'Interessato è liberamente prestato ed è revocabile in ogni momento senza che ciò comporti alcun svantaggio o pregiudizio e senza pregiudicare la liceità del trattamento basata sul consenso prestato prima della revoca.
Le istanze relative all'esercizio di tali diritti potranno essere presentate al Centro di Sperimentazione e/o al Promotore contattandolo ai recapiti sopra riportati.

Infine, ove l'Interessato ritenga che il trattamento dei suoi dati personali avvenga in violazione di quanto previsto dal Regolamento (UE) 2016/679 o del D.lgs. 196/03 s.m.i., ha il diritto di proporre reclamo al Garante, come previsto dall'art. 77 del Regolamento citato, o di adire le opportune sedi giudiziarie (art. 79 del Regolamento).

Ulteriori informazioni inerenti il progetto di ricerca
Ulteriori informazioni inerenti il progetto di ricerca in oggetto possono essere richieste a:
Prof. V. M. Ranieri
U.O Anestesiologia e Terapia Intensiva Polivalente - Azienda Ospedaliera Universitaria di Bologna, Policlinico di Sant'Orsola, Via Massarenti, 9 – 40138 Bologna,
Tel. 051.214.3268, Fax 051.636.4333.
m.ranieri@unibo.it

Consenso al trattamento dei dati personali e particolari

Versione del 12/11/2019

**TITOLO STUDIO:**	"Rimozione extracorporea di anidride carbonica per il trattamento della BPCO riacutizzata: studio clinico randomizzato e controllato"
**PROTOCOLLO No:**	ORION

Il/la sottoscritto/a____

nato/a a____ il_____________________________

e residente a____________________ in Via____

telefono____

☐ **in qualità di diretto Interessato**

oppure in qualità di

☐ **rappresentante legale (*)** ____

(specificare se esercente la responsabilità genitoriale, tutore, curatore, amministratore di sostegno)

del paziente ____

(specificare il nominativo del paziente per il quale si rilascia il consenso)

nato/a a____ il_____________________________

residente a ____

Via/Piazza____ telefono -_____________________

DICHIARA

- di aver ricevuto e preso attenta visione del documento "Foglio informativo-partecipazione allo studio";
- di aver preso attenta visione delle "Informazioni sul trattamento dei dati personali e particolari" sopra riportata e di averne compreso i contenuti e le ulteriori informazioni ottenute in merito dallo Sperimentatore e/o dagli altri Collaboratori;

Ai sensi delle disposizioni del Regolamento (UE) 2016/679 e del D.Lgs. 196/2003 s.m.i., lette le "Informazioni sul trattamento dei dati personali e particolari" sopra riportate, il/la sottoscritto/a

☐ **Acconsente** ☐ **Non acconsente**

al trattamento dei propri dati - NECESSARIO ai fini della partecipazione allo studio di cui trattasi e al suo svolgimento per le finalità e nei modi di cui al punto (A) [*partecipazione allo studio e relative operazioni ed attività connesse*]

☐ **Acconsente** ☐ **Non acconsente**

alla conservazione e all'ulteriore utilizzo da parte del PROMOTORE - NON NECESSARIA ai fini della partecipazione allo studio di cui trattasi e al suo svolgimento – dei dati personali dell'Interessato per le finalità e nei modi di cui al punto (B) [*conservazione dei dati e dei campioni biologici per future attività di ricerca*]

☐ **Acconsente** ☐ **Non acconsente**

alla conservazione e all'ulteriore utilizzo da parte del CENTRO DI SPERIMENTAZIONE - NON NECESSARIA ai fini della partecipazione allo studio di cui trattasi e al suo svolgimento – dei dati personali dell'Interessato per le finalità e nei modi di cui al punto (B) [*conservazione dei dati e dei campioni biologici per future attività di ricerca*]

☐ **Acconsente** ☐ **Non acconsente**

alla comunicazione di cui al punto (I) - NON NECESSARIA ai fini della partecipazione allo studio - dei dati dell'Interessato al suo medico curante affinché sia informato della partecipazione allo studio in parola [*comunicazione al proprio medico curante*]

☐ **Acconsente** ☐ **Non acconsente**

alla ricezione delle informazioni di cui al punto (II) - NON NECESSARIA ai fini della partecipazione allo studio – affinché sia informato di eventuali risultati e/o notizie inattese che riguardano l'Interessato *[comunicazione di risultati o notizie inattese]*

Data **Firma**

________________ ____________________________________

Nome del/la paziente ____

(in stampatello)

DIPARTIMENTO DI SCIENZE MEDICHE E CHIRURGICHE

DICHIARAZIONE DEL MEDICO O ALTRO PROFESSIONISTA SANITARIO CHE HA FORNITO LE “INFORMAZIONI SUL TRATTAMENTO DEI DATI PERSONALI E PARTICOLARI” AL PAZIENTE

DICHIARO

- di aver fornito al/alla paziente informazioni complete e spiegazioni dettagliate circa la natura, le finalità, le procedure e la durata dello studio di cui trattasi e dei relativi trattamenti di dati personali
- di aver fornito al/alla paziente copia datata e firmata del presente documento contenente le “Informazioni sul trattamento di dati personali e particolari” e il modulo per il “Consenso al trattamento dei dati personali e particolari” e di aver acquisito il consenso del rappresentante legale identificato tramite il documento

____
(indicare estremi del documento)

Nome del Medico o altro Professionista Sanitario ____________________________
(in stampatello)

Data ______________ **Sanitario**

Firma del Medico/Professionista

____

ALMA MATER STUDIORUM - UNIVERSITÀ DI BOLOGNA
SEDE AMMINISTRATIVA C/O AZIENDA OSPEDALIERO-UNIVERSITARIA – POLICLINICO S. ORSOLA–MALPIGHI
VIA MASSARENTI 9, PADIGLIONE 11 - 40138 BOLOGNA - ITALIA
